# Perturbation Predictability Can Influence the Long-Latency Stretch Response

**DOI:** 10.1371/journal.pone.0163854

**Published:** 2016-10-11

**Authors:** Christopher J. Forgaard, Ian M. Franks, Dana Maslovat, Romeo Chua

**Affiliations:** School of Kinesiology, University of British Columbia, Vancouver, Canada; Ehime University Graduate School of Medicine, JAPAN

## Abstract

Perturbations applied to the upper limbs elicit short (M1: 25–50 ms) and long-latency (M2: 50–100 ms) responses in the stretched muscle. M1 is produced by a spinal reflex loop, and M2 receives contribution from multiple spinal and supra-spinal pathways. While M1 is relatively immutable to voluntary intention, the remarkable feature of M2 is that its size can change based on intention or goal of the participant (e.g., increasing when resisting the perturbation and decreasing when asked to let-go or relax following the perturbation). While many studies have examined modulation of M2 between passive and various active conditions, through the use of constant foreperiods (interval between warning signal and a perturbation), it has also been shown that the magnitude of the M2 response in a passive condition can change based on factors such as habituation and anticipation of perturbation delivery. To prevent anticipation of a perturbation, most studies have used variable foreperiods; however, the range of possible foreperiod duration differs between experiments. The present study examined the influence of different variable foreperiods on modulation of the M2 response. Fifteen participants performed active and passive responses to a perturbation that stretched wrist flexors. Each block of trials had either a short (2.5–3.5 seconds; high predictability) or long (2.5–10.5 seconds; low predictability) variable foreperiod. As expected, no differences were found between any conditions for M1, while M2 was larger in the active rather than passive conditions. Interestingly, within the two passive conditions, the long variable foreperiods resulted in greater activity at the end of the M2 response than the trials with short foreperiods. These results suggest that perturbation predictability, even when using a variable foreperiod, can influence circuitry contributing to the long-latency stretch response.

## Introduction

Mechanical perturbations applied to joints in the upper limbs elicit stereotyped responses in the electromyographic (EMG) recording of the stretched muscle. The first event, termed the short-latency (M1) response, is produced by a monosynaptic spinal reflex pathway and appears in the EMG recording ~25–50 ms after the onset of muscle stretch [1]. Immediately following M1, occurring between ~50–100 ms, is a second event termed the long-latency (M2) stretch response [2]. M2 is generated (at least in part) by a transcortical pathway involving the primary sensory and motor cortices [3–10]. While M1 is usually resistant to voluntary intervention, a remarkable feature of M2 is that it can be modulated based on the goal or intent of the subject. For instance, when instructed to counteract the perturbation, M2 increases in magnitude thus aiding to offset the imposed load. This modulation is believed to result from pre-setting excitability of the contributing neural circuitry [2, 5]; although alternative explanations have also been provided [11–14].

While numerous studies have examined the influence of intentional set, other factors such as habituation [15], event predictability [16], and temporal anticipation of the perturbation [15, 17] have also been shown to influence the M2 response. For example, Rothwell et al. [15] reduced the temporal uncertainty of perturbation delivery, by either cueing perturbation onset with a weak electric stimulus, or having participants self-deliver the perturbation by pressing a button. Stretch responses from these predictable conditions were compared with a less predictable condition, where the perturbation was delivered every 5 seconds. M1 did not change across conditions; however M2 was reduced in magnitude on trials with increased temporal certainty. Similar results were observed from an experiment where participants were asked to actively respond by either “letting go” or “opposing” the perturbation [17]. Like the study by Rothwell and colleagues [15], predictable perturbations were self-delivered; however in the experiment by Goodin and Aminoff [17], unpredictable perturbations were given randomly every 8–116 seconds (i.e., at very unpredictable intervals). Comparable to previous findings, M1 was not affected by either intentional set or perturbation predictability. Of particular interest was the finding that M2 was smaller following predictable perturbations, and this occurred for both the “let-go” as well as the “oppose” conditions.

The experiments discussed above highlight findings from the extremes of temporal predictability. In order to prevent anticipation of perturbation delivery, many studies examining M2 have used variable intervals between the warning signal and perturbation (i.e., foreperiod), or between subsequent perturbations (if no warning signal was provided). However, the range of intervals used differs considerably between studies. For example, a recent study by Pruszynski et al. [5] employed short variable foreperiods of 1–1.5 seconds; we [18] have previously used foreperiods ranging from 2.5–3.5 seconds; while Manning et al. [11] used long variable foreperiods of 3–10 seconds. It remains unclear whether the range of possible variable foreperiod length influences the circuitry contributing to the M2 response. We were concerned that a less predictable (i.e., long) foreperiod may result in a larger M2 response on passive trials. This could reduce the potential for any further increases in excitability when participants actively respond to the perturbation and may account for why some studies have not observed goal-dependent modulation of the M2 response (e.g., [11]).

The purpose of the present study was to examine whether the predictability produced by the length of variable foreperiod can influence the M2 response. We compared blocks of short (2.5–3.5 seconds; high predictability) and blocks of long (2.5–10.5 seconds; low predictability) variable foreperiods where participants were instructed to either not intervene (passive) or to compensate (active) for the perturbation as quickly as possible. It was expected that M2 would be larger on active trials (compared to passive), but of primary interest was whether M2 could also be modulated as a function of the range of foreperiod durations.

## Materials and Methods

Fifteen right-handed healthy participants (8 male, 7 female; mean age of 22 +/- 3 years) participated in an experiment lasting approximately 90 minutes. Informed written consent was collecting prior to each testing session and the procedures were approved by the University of British Columbia behavioural ethics board.

## Experimental Setup

Participants sat in a height-adjustable chair with right arm secured to a manipulandum that allowed movement to occur in the horizontal plane about the wrist joint. The elbow was flexed at 100 degrees and hand semi-supinated with the wrist joint aligned to the rotational axis of the manipulandum. A torque motor (Aeroflex TQ 82W-1C) was connected to the manipulandum and a metal handle attached to the motor shaft was positioned near the metacarpophalangeal joints. Foam stops were tightened on either side of the wrist to prevent lateral movement and custom-molded thermoplastic was tightened around the hand of each participant allowing movement to occur without the fingers grasping the metal handle. An oscilloscope was placed on a table ~1 m in front of the manipulandum and provided continuous feedback of wrist position and a LED lightbox was placed on top of the oscilloscope.

## Task and Stimuli

The starting position for each perturbation trial was 10 degrees of wrist flexion, a point defined visually on the oscilloscope by arrows. Trials began with the torque motor ramping up (over 500 ms) to a small extension preload of 0.25 newton metres (Nm). To resist the preload and keep the wrist at the home position, participants generated a slight contraction in wrist flexors. A warning signal was generated by the lightbox when the preload reached its peak. If the light turned red participants were instructed to “not intervene with the perturbation” (i.e., passive, do not intervene condition: DNI), while if the light was green participants were instructed to “flex the wrist as quickly as possible following the perturbation” (i.e., active condition: ACT). The foreperiod was terminated with a large wrist extension perturbation of 1.5 Nm lasting 150 ms. Following the perturbation, the preload level of torque remained for 450 ms.

Differences in foreperiod duration can influence reaction time (RT; e.g., [19]); however, due to preceding reflexive responses, RT cannot be determined on a trial-by-trial basis in a perturbation paradigm (for more detail see [11, 18]). We included two separate conditions to determine whether RT differences between the two foreperiod ranges may have occurred in our perturbation conditions. Procedures were identical to those described above and included all four types of trials (ACT and DNI, Short and Long variable foreperiods); however the imperative stimulus was an 80 dB auditory tone (no perturbations occurred in these blocks) and the starting position was 30 degrees of wrist extension (approximately where the perturbation displaced the wrist; e.g., [18]). While this was not meant to provide an estimate of RT in the perturbation conditions, the auditory cue conditions were meant to provide evidence that RT differences were present in the foreperiod ranges that we used and that this would presumably also be present during perturbation trials (albeit at a different response latency due to faster RT on perturbation trials).

Participants performed 4 blocks of 50 experimental trials (200 trials total) in which the variable foreperiod between the warning signal and the perturbation (or auditory imperative signal) was short (2.5–3.5 seconds: SFP) or long (2.5–10.5 seconds: LFP). Prior to commencing the experimental blocks, participants were provided 10 ACT and 10 DNI practice trials for each foreperiod length. Within an experimental block, 25 ACT and 25 DNI trials were randomly interleaved. To control the inter-stimulus rate (between trials conducted in different blocks), a minimum interval between onset of each trial was 15 seconds. Experimental block order was randomized across participants and separated by a 5 minute rest period.

## Data Collection, Reduction, and Analysis

Surface EMG data were collected from the right wrist flexor (FCR) and extensor (ECR) carpi radialis muscles using bipolar preamplified silver/silver chloride surface electrodes connected to an external amplifier (Model 544, Therapeutics Unlimited Inc., Iowa City, IA). EMGs were amplified at 2-4K and bandpass filtered from 30–1000 Hz. Signals were sampled at 2 kHz using a 1401Plus data acquisition system and a computer running Spike2 (CED, Cambridge, UK). Offline data analysis was accomplished using Spike2 and custom-written LabVIEW (National Instruments, Austin, TX) software.

EMG data were baseline corrected and full-wave rectified. A 700 ms epoch (200 ms pre to 500 ms post) was defined around each imperative signal. Visual inspection was conducted on individual trial data from FCR, ECR, and the displacement profile to ensure correct performance. Reasons for trial exclusion included responding before the imperative signal (i.e., false starts), not responding on an ACT trial, or responding on a DNI trial. Of the 3500 experimental trials collected, 2.6% were omitted due to error.

For the perturbation conditions, individual participant ensemble averages (of ~25 trials) were used to obtain stretch response onset and offset times (see [4, 11, 18]). Average baseline EMG and standard deviation (SD) were calculated from -200 to 0 ms relative to perturbation onset. M1 onset was determined as the point at which activity first increased 2 SD above baseline levels. Due to overlap between the end of M1 and onset of M2, we could not use the same criteria to mark M2 onset, therefore this point was defined as the trough in activity (occurring around 50 ms). With voluntary activity overlapping onto the end of M2 in the ACT conditions, M2 offset was only marked from DNI conditions (where no voluntary response was present). This was determined as the first decrease in activity below 2SD above baseline following M2 onset.

For the perturbation conditions, integrated values from the wrist flexor EMG data were analyzed in four 25 ms epochs relative to perturbation onset (on a trial-by-trial basis). The first time period was used to examine baseline EMG, occurring 25 ms prior to the onset of the perturbation. The second epoch contained the M1 response, 25–50 ms post-perturbation. While the M2 response in wrist flexors typically occurs between 50 and 100 ms, a current hypothesis in the literature is that activity during this interval is not generated by a single pathway; rather multiple spinal and supra-spinal circuits make contributions (e.g., [20, 21, 22]). Furthermore, on ACT trials, the voluntary response (sometimes referred to as a triggered reaction) may also superimpose onto the latter half of the M2 response (e.g., [11, 12, 18]). Therefore, taking a similar approach to other authors (e.g., [12, 18, 23]), we have divided the M2 period into two parts, M2a (50–75 ms) and M2b (75–100 ms).

The mean integrated activity from the M1 epoch (across the four perturbation conditions) was used to normalize the integrated EMG data for each participant. A value of 1.0 corresponds to integrated EMG values obtained in the M1 epoch. We were also interested in whether normalized integrated activity within each epoch changed as a function of foreperiod length on a given trial. We computed a Pearson correlation coefficient from each epoch versus the time at which the perturbation occurred. In addition, we calculated the raw peak M1 (25–50 ms) and peak M2 values (50–100 ms) from each trial. These values are presented in Table 1. For the auditory conditions, mean baseline (from -200 ms to the imperative signal) and standard deviation (SD) of FCR activity was calculated on each ACT trial. RT was determined as the first point that activity exceeded 3 SD above baseline and remained above this level for at least 20 ms. Onset marker positions were verified visually and adjusted only on the rare occasion where the algorithm resulted in an obvious error. DNI trials from these conditions were analyzed to ensure participants successfully withheld a voluntary response on these trials.

**Table 1:** Mean values (and inter-participant standard deviations) from our dependent measures of interest and omnibus ANOVA results. Onset/Offset data are in milliseconds. M2 peak values are in millivolts. Epoch data are normalized to each participant’s mean integrated M1 values (normalized units).

	Condition				Omnibus Test											
	Short Foreperiod		Long Foreperiod		Foreperiod				Intentional Set				Foreperiod × Set			
	DNI	ACT	DNI	ACT	*df*	*F*	*p*	$\eta_{p}^{2}$	*df*	*F*	*p*	$\eta_{p}^{2}$	*df*	*F*	*p*	$\eta_{p}^{2}$
M1 Onset (ms)	27.6 (2.4)	27.3 (2.2)	27.7 (2.9)	27.4 (2.5)	1, 14	0.07	.79	< .01	1, 14	3.15	.098	.18	1, 14	0.01	.96	< .01
M2 Onset (ms)	49.8 (3.2)	50.0 (3.3)	49.7 (3.8)	49.5 (3.2)	1, 14	0.76	.40	.05	1, 14	0.01	.96	< .01	1, 14	0.57	.46	.04
M2 Offset (ms)	93.4 (7.4)	-	97.7 (6.8)	-	14	*t* 5.03	< .001	-	-	-	-	-	-	-	-	-
M1 Peak (mV)	0.18 (0.10)	0.19 (0.11)	0.16 (0.08)	0.17 (0.09)	1, 14	2.64	.13	.16	1, 14	2.15	.16	.13	1, 14	0.07	.80	< .01
M2 Peak (mV)	0.48 (0.27)	0.71 (0.38)	0.51 (0.24)	0.72 (0.39)	1, 14	0.46	.51	.03	1, 14	17.90	.001	.56	1, 14	0.33	.58	.02
Baseline Epoch (NU)	0.32 (0.15)	0.31 (0.14)	0.29 (0.14)	0.31 (0.15)	1, 14	0.61	.45	.04	1, 14	0.03	.88	< .01	1, 14	4.11	.062	.23
M1 Epoch (NU)	1.01 (0.13)	1.08 (0.15)	0.92 (0.15)	0.98 (0.16)	1, 14	2.00	.18	.13	1, 14	3.25	.093	.19	1, 14	0.01	.91	< .01
M2a Epoch (NU)	2.90 (1.73)	3.48 (2.01)	2.81 (1.09)	3.51 (1.82)	1, 14	0.13	.91	< .01	1, 14	10.14	.007	.42	1, 14	0.48	.50	.03
M2b Epoch (NU)	1.89 (1.50)	4.50 (3.06)	2.36 (1.82)	4.55 (3.34)	1, 14	1.70	.21	.11	1, 14	20.38	< .001	.59	1, 14	6.68	.022	.32

RT values from the auditory conditions were analyzed using a paired samples *t*-test comparing ACT trials from the 2 Foreperiods (SFP, LFP). A paired samples *t*-tests was also used to compare M2 offset between the two perturbation DNI conditions. For the perturbation conditions, M1/M2 onsets and the integrated normalized EMG data from each of the predefined epochs (Background, M1, M2a, M2b) were analyzed using a 2 Foreperiod (SFP, LFP) × 2 Intentional Set (DNI, ACT) repeated measures analysis of variance (ANOVA). We used partial eta squared ($\eta_{p}^{2}$) to express effect size and significant Foreperiod × Intentional Set Interactions were interpreted using simple main effects analysis. The level of statistical significance for these tests was set at *p* = .05. For the correlation analysis between trial-by-trial foreperiod length and normalized integrated EMG activity within each epoch, Pearson *r* values underwent a Fisher *r* to *Z* transformation. The transformed values were statistically analyzed using One-Sample *t*-tests to determine whether any values differed significantly from zero. To correct for four comparisons (DNI SFP, DNI LFP, ACT SFP, ACT LFP) within each epoch, the level of statistical significance for these tests was set a *p* = .0125.

## Results

**Fig 1:**
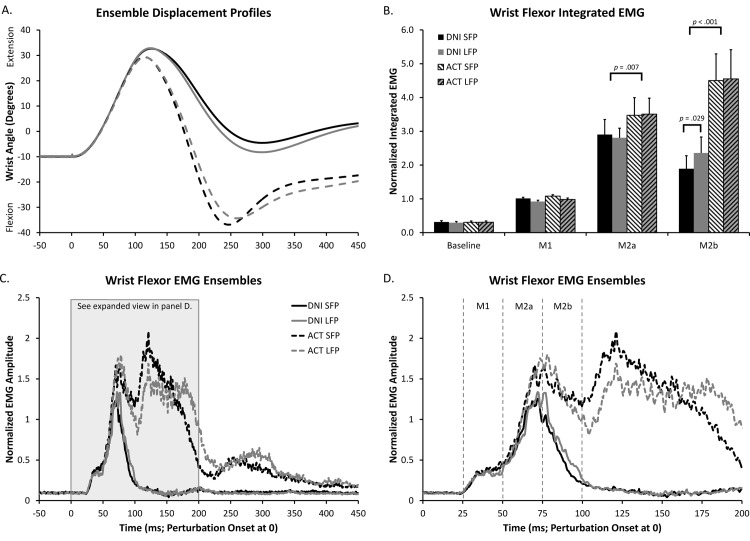
Group (N = 15) displacement and wrist flexor EMG data. A. Ensemble wrist displacement in degrees. B. Normalized integrated wrist flexor EMG values for the epochs of interest. Values were normalized to each participant’s mean integrated EMG activity in the M1 epoch. C. Normalized rectified wrist flexor EMG along same time scale as A. Values were normalized to each participant’s mean peak M1. D. Same as C, but zoomed-in to focus on the time periods of interest.

Group wrist displacement profiles, wrist flexor EMG ensembles, and integrated EMG values from each epoch of interest are presented in Fig 1. A statistical summary is also provided in Table 1.

When participants responded to an auditory signal, RT values from the SFP block (141.1 ms) were shorter than values obtained in the LFP block (154.5 ms), a difference that approached statistical significance (*t*(14) = 2.14, *p* = .051).

The perturbation produced two clear responses in wrist flexors. The first response (M1) had a mean onset latency of 27.5 ms, a value which did not differ across the four conditions. The second response (M2) had a mean onset of 49.8 ms and also was not different between conditions. The offset of M2 could only be determined from the two DNI conditions; this value occurred earlier (*t*(14) = 5.03, *p* < .001) for the SFP block (93.4 ms) compared to the LFP block (97.7 ms).

Analyzing integrated area of the baseline and M1 epochs revealed no main effects or interactions between conditions (see Table 1 for mean values and a statistical summary). By contrast, differences were found for the epochs containing the long-latency stretch response (M2a/M2b). Analysis of the M2a period revealed a significant main effect of Intentional Set, (*F*(1, 14) = 10.14, *p* = .007, $\eta_{p}^{2}$ = .42), indicating that activity on ACT trials was significantly larger than on DNI (see Fig 1). A main effect of Intentional Set was also observed for the M2b epoch, (*F*(1, 14) = 20.38, *p* < .001, $\eta_{p}^{2}$ = .59); however this was superseded by a significant Intentional Set × Foreperiod Interaction (*F*(1, 14) = 6.68, *p* = .022, $\eta_{p}^{2}$ = .32). Simple main effects analysis revealed increased M2b activity for the DNI LFP (2.36 Normalized Units: NU) condition compared to DNI SFP (1.89 NU; *p* = .029) (see Fig 1, Solid Grey vs. Solid Black profiles). Significant foreperiod differences between the two ACT conditions (*p* = .83) were not observed; nevertheless both ACT conditions remained significantly larger than DNI.

**Fig 2:**
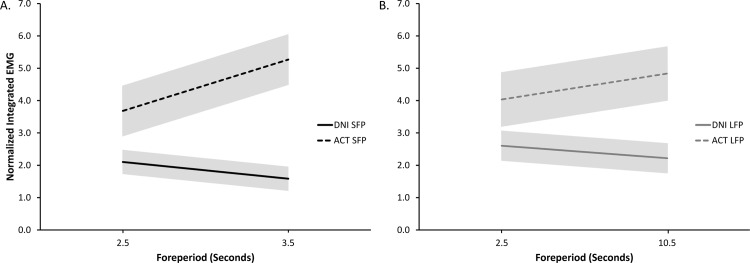
M2b group mean (and standard error) lines of best fit for the normalized integrated wrist flexor EMG values as a function of trial-by-trial foreperiod length. DNI conditions: solid lines. ACT conditions: dashed lines. A. Short foreperiod (2.5–3.5s) conditions. B. Long foreperiod (2.5–10.5s) conditions. For ACT conditions, as the foreperiod aged, M2b values tended to increase. For the DNI conditions, M2b values decreased with the aging foreperiod.

The trial-by-trial correlation analysis between foreperiod length and integrated activity revealed no significant correlations for the baseline, M1, or M2a epochs (all *r* values -.09 to.11; all *p* values > .06. This suggested that activity during these time periods was not modulated as a function of foreperiod length on a given trial. By contrast, analysis of the M2b epoch revealed significant, or nearly significant (critical *p* = .0125) correlation values for each condition (see Fig 2 for mean lines of best fit from the M2b epoch). The ACT conditions showed a small positive trend (SFP: *r* = 0.21, *t*(14) = 4.29. *p* = .001; LFP: *r* = 0.18, *t*(14) = 3.95, *p* = .001), which was likely due to an aging foreperiod effect on RT (i.e., more voluntary superimposition because RT of the voluntary response was expected to be shorter as the foreperiod aged). A small negative trend was observed for the DNI conditions (SFP: *r* = -0.19, *t*(14) = -3.16. *p* = .007; LFP: *r* = -0.15, *t*(14) = -2.68. *p* = .018). This we believe was due to the participants being better able to anticipate perturbation delivery as they neared the end of the potential foreperiod range. Importantly, within a given Intentional Set (ACT or DNI), similar aging foreperiod effects were found between the two foreperiod conditions (SFP and LFP).

## Discussion

The present study investigated whether the temporal predictability of a limb perturbation can influence the long-latency stretch response. While previous investigations have compared the extremes of temporal (un)certainty [15, 17], we made comparisons between two variable foreperiods of different potential duration. Replicating previous research (e.g., [2, 4, 5, 18]), M2 activity was increased on trials where participants were instructed to compensate for the perturbation compared to passive trials where participants were asked not to intervene. The main finding from this study was that even though variable foreperiods are often used to prevent anticipation of a perturbation, the range of possible foreperiod duration still influenced the M2 response on passive trials. Specifically, the more predictable (short) variable foreperiod resulted in reduced M2b activity compared to the less predictable (long) variable foreperiod condition. Our study also revealed “aging foreperiod” effects during the M2b epoch for all perturbation conditions; these effects provide further evidence that temporal predictability of a mechanical perturbation can influence the long-latency stretch response.

Integrated area within a predefined epoch is influenced by the amplitude as well as the duration of activity. Analysis of our stretch response timing data revealed no differences between the conditions with regards to the onset of M1 or M2. We did however find an unexpected difference when examining M2 offset. Specifically, the duration of M2 was ~4 ms shorter for the SFP DNI condition compared to the LFP DNI condition. By contrast, peak M2 values did not significantly differ between DNI conditions (see Table 1). Therefore the reduced integrated M2b activity from the SFP block appeared to result from a shorter M2 duration, as opposed to a smaller M2 peak. The previous investigations examining perturbation predictability [15, 17] only reported integrated EMG records, so it is unclear whether differences between conditions resulted from M2 timing and/or amplitude differences.

A majority of studies examining the M2 response have used perturbations which rapidly stretched a muscle of interest, but muscular responses occurring over a similar time-course have also been observed following the sudden application of load in a precision grip task. These latter responses are believed to be elicited by cutaneous tactile afferents in the fingers. Kourtis et al. [24] compared the predictability of a sudden load perturbation on the size of the long-latency response in a precision grip task. Participants were asked to hold an object between the right thumb and index finger and to not let it slip following application of a downward load. In one condition the load was given predictably every 2 seconds, whereas in another condition the load was randomly applied between 0.7 and 4.3 seconds. Similar to the stretch response findings of the present study, Kourtis et al. found that the long-latency response was reduced following the predictable load change. These authors reported an amplitude difference between the two conditions, but from their figures it appeared that duration of the long-latency response was also shorter following the predictable perturbation.

A long-standing debate in the stretch response literature is whether goal-dependent modulation of activity during the M2 response epochs are produced from changes in excitability of the underlying circuitry (e.g., [2, 5]) or is an artifact of the voluntary or triggered response superimposing onto the stretch response (e.g., [11, 12–14]). An early study which argued for the latter mechanism also made comparisons using foreperiods of varying stimulus predictability [14]. Because trials with more predictable stimulus onsets can have shorter RTs (e.g., [19]), Rothwell and colleagues hypothesized these conditions would show the greatest M2 period modulation between “resist” and “let-go” instructions. Indeed, the greatest change to M2 was observed from conditions where perturbation onset was most predictable. However, a finding not mentioned by Rothwell et al. (but evident from their figures and pointed out by other authors; e.g., [25]) were differences between the “let-go” conditions. M2 activity was reduced (nearly in half) when participants performed in the most predictable stimulus condition. Because there was no overlapping voluntary response on “let-go” trials, but changes to M2 were still observed, this supports the hypothesis that changes in reflex circuit excitability can influence the M2 response. The “let-go” condition has however been criticised because it may not represent an unmodified stretch response (e.g., [13, 26]); the instruction implies participants actively relax upon receiving the perturbation [26]. In our study we observed M2 differences using the passive “do-not intervene” instruction. Like the “let-go” instruction, the DNI condition is also not confounded by a superimposed voluntary response, therefore the M2b modulation that we observed likely reflected differences in contributions from the underlying M2 circuitry as opposed to a superimposed voluntary response.

Pathways mediating the long-latency stretch response have been extensively investigated since the 1970’s. Early work focused on whether M2 was generated by spinal circuitry (e.g., [27, 28]), or a longer transcortical pathway (e.g., [3, 10, 29]). A current proposition is that M2 is not produced by a single pathway; rather it receives contributions from multiple spinal and supra-spinal pathways [20–22]. Primary motor cortex [5, 7, 8] and the cerebellum [30, 31] have been highlighted as important structures involved in modulation of the M2 response. Interestingly, both structures have also been implicated in temporal anticipation and predictability of sensory stimuli (e.g., [17, 32, 33, 34]). Lee and Tatton [29] reported the presence of two reflex peaks in the M2 period; the first was referred to as M2 and the second was called M3. These authors proposed that M2 was produced via primary sensory and motor cortex and M3 traversed the cerebellum prior to engaging motor cortex. Although we did not observe two distinct reflex peaks during the M2 epochs, the M2b epoch may correspond closely to the M3 response reported by Lee and Tatton. Because both cerebellar and motor cortex circuitry potentially make contributions to the M3 response [29], and both the cerebellum [32, 33] and motor cortex [17, 34] has been shown to be involved in temporal anticipation, it is plausible that both structures may contribute to the M2b differences observed between our DNI conditions.

When participants actively respond to a perturbation, voluntary RTs have been observed at latencies as short at ~70 ms [4, 11–13] making it difficult to interpret changes to the latter portion of M2 for ACT conditions (see [11, 18]). Even though we did not observe significant foreperiod differences between the two ACT conditions, this does not necessarily preclude the possibility that differences in M2 and/or voluntary response overlap may have occurred. For example, RTs were expected to be shorter for trials from the SFP block (e.g., [19]) and indeed, the voluntary response appeared earlier and larger for this condition (Fig 1C and 1D: dashed black profile) compared to the LFP block (dashed grey profile). This would lead one to expect increased M2b activity for the SFP block, a result of greater voluntary response superimposition. By contrast, if differences in the M2 response itself occurred (e.g., duration, similar to the DNI conditions), we would predict trials from the LFP block to have a longer M2 response compared to trials from the SFP block. In other words, poor perturbation anticipation was expected to increase duration of the M2 response and result in a later voluntary response onset (and hence less voluntary response superimposition onto M2). By contrast, enhanced anticipation (SFP condition) may result in reduced M2 activity, but also shorter voluntary response latency (and more overlap onto M2). The net result of these two factors would be an M2b period of similar integrated area between ACT conditions differing in predictability of the perturbation.

Although our study was not specifically designed to examine the effects of foreperiod aging on modulation of perturbation elicited stretch responses, a correlation analysis of trial-by-trial foreperiod length and activity in each predefined epoch revealed interesting findings. The duration of the foreperiod on a given trial had no influence over activity during the baseline, M1, or M2a epochs. By contrast, all perturbation conditions showed an effect of trial-by-trial foreperiod length on activity during the M2b epoch (see Fig 2). The active conditions showed increased M2b activity as the duration of the foreperiod increased. This was likely the result of a typical aging foreperiod effect on RT of the voluntary response, with RT decreasing as the foreperiod aged and imperative signal delivery became imminent (e.g., [35]). The opposite trend was observed for DNI conditions with M2b activity decreasing as the foreperiod progressed. The reduced M2b activity may have resulted from a similar temporal anticipation mechanism responsible for our main DNI findings (i.e., more predictable perturbations resulting in reduced M2b activity).

In summary, while previous investigations have shown self-delivered [15, 17] and temporally cued [15] perturbations can modulate the M2 response, the novel finding from our study was that the M2 response was also sensitive to small differences in the range of potential foreperiod duration. In our study, and the previous studies that have shown temporal perturbation predictability can reduce M2 activity, the direction of the perturbation was always known in advance [15, 17]. A recent study [36] suggested that temporal predictability may actually have the opposite effect on the M2 response when perturbation direction is not precued. Therefore, while we do not advocate researchers use only short or long variable foreperiods, we do believe that it is important that considerations be given to the effect that foreperiod variation can have on the long-latency stretch response.
